# A consolidated framework for implementation research (CFIR) informed exploration of a primary care intervention to support deprescribing for problematic polypharmacy in older adults living with frailty (DEPPLOY) in England: a qualitative study

**DOI:** 10.1007/s11096-026-02140-0

**Published:** 2026-04-27

**Authors:** Liz Breen, Amrit Daffu-O’Reilly, Aliya Darr, Jonathan Benn, Beth Fylan, Jonathan Silcock, George Peat, D. K Theo Raynor, Duncan R. Petty, Syed Tabish Zaidi, Alison Bravington, Nazreen Butt, David P. Alldred

**Affiliations:** 1 University of Bradford, Bradford, UK; 2 Keele University, Newcastle, UK; 3 University of Leeds, Leeds, UK; 4 NIHR Yorkshire and Humber Patient Safety Research Collaboration, Bradford Teaching Hospitals NHS Foundation Trust, Bradford, UK; 5 University of Northumbria, Newcastle, UK; 6 University of Tasmania, Hobart, Australia

**Keywords:** CIFR, Deprescribing, Frailty, Implementation science, Polypharmacy, Primary care, Structured medication reviews

## Abstract

**Introduction:**

The incorporation of deprescribing into structured medication reviews (SMR) is a patient-centred and cost-effective practice in primary care settings. Pharmacists have a key role in deprescribing among older people to improve quality of life and reduce adverse events, but patients and healthcare professionals may be resistant to reducing their medication, and evidence around how deprescribing happens in practice is currently lacking. This study explores the implementation of a co-designed intervention in a single English General Practice (UK).

**Aim:**

To explore key stakeholders’ perceptions of a co-designed primary care intervention to involve patients and their families in deprescribing and its broader intervention context, guided by the Consolidated Framework for Implementation Research (CIFR).

**Method:**

Qualitative semi-structured interview study with a purposive sample of staff, and patients with frailty, to explore perceptions of the deprescribing initiative. Data were analysed using an a priori framework structured by the domains of the CIFR.

**Results:**

Interviews (24 in total) were conducted with 13 staff involved in delivering the intervention and 5 patients living with frailty who completed a structured medication review with recommended medication changes. Key factors (mapped to CFIR domains) included: an imperative for formal training around the intervention delivery (Inner setting), engagement with SMR delivery linked to payment through national agendas (Outer setting); the importance of the fit of the intervention with existing processes around prescribing practice and infrastructure (Inner setting); increased understanding of the aims of deprescribing among patients and recognition of the extended pharmacist role in primary care deprescribing (Individuals); recognition that the successful delivery of the intervention was a team effort (Implementation process).

**Conclusion:**

Structured medication reviews are a suitable mechanism to discuss and make deprescribing decisions as part of a shared consultation. Resources which support the patient through the deprescribing process can engage patients and promote greater satisfaction with service delivery. Operationally, staff can also benefit from tools which facilitate greater understanding of the process and fit within their usual practice plus improving patient care and saving medication costs. Barriers and facilitators to implementation success should be noted and addressed for upscaling and process sustainability.

**Supplementary Information:**

The online version contains supplementary material available at 10.1007/s11096-026-02140-0.

## Impact statements

- Pharmacists are recognized for their extended professional role in UK primary care and are optimally placed to lead deprescribing initiatives.
- A robust tool to identify patients who would benefit from deprescribing consultations is highly beneficial to primary care practice.
- Host sites with resource richness and supporting infrastructure, in the form of dedicated clinics, are likely to succeed in sustainable deprescribing by devoting time to developing older people’s understanding of stopping medicines during a Structured Medication Review (SMR).
- Patients are empowered to have informed shared consultations and manage their medicines more confidently when prepared to discuss their medicines in SMRs and when they have a record of their consultation.
- Primary care sites may need additional guidance, training and resources to embed this intervention into routine processes.

## Introduction

Frailty in older people, defined as the ‘distinctive health state related to the ageing process in which multiple body systems gradually lose their in-built reserves’ [1], brings an increased risk of adverse health outcomes, including reduced muscle strength and increased fatigue. As frailty progresses, the ability to ‘bounce back’ from health-related events diminishes, and even minor health issues can become major concerns. In the UK, approximately 10% of people over 65 live with frailty, rising to 25–50% among those over 85 [2].

Frailty is also associated with adverse reactions to medicines [3], particularly polypharmacy (the use of multiple medications), which increases the likelihood of unwanted events such as cognitive decline and falls. Older adults often take medicines with a high anticholinergic burden [4–6], which complicates medication management. As frailty increases, medication responses become less predictable, making medicines optimisation a priority [7–9].

Medicines optimisation includes deprescribing – stopping medicines that are no longer needed, or where the risks outweigh the benefits [10]—to improve quality of life and reduce adverse events [11]. Structured medication reviews (SMRs) provide an opportunity for shared decision-making with patients at risk of harm [12]. In England, SMRs have been a service requirement for primary care networks (PCNs) since 2021/22 with payment by activity [13], focusing discussion on patient’s individual needs, preferences and circumstances [12], although how they deliver this agenda is contested [14].

Deprescribing is the process of tapering or stopping medicines to minimise unnecessary polypharmacy and improve medicine-related outcomes [15]. The process of deprescribing has been demonstrated to improve medication-related outcomes such as medication appropriateness and reduction in medication burden; however, there is limited evidence of effects on patient-orientated outcomes such as adverse events, hospital admissions and mortality [16]. A reduction in polypharmacy and medication burden may potentially have positive effects on adherence [17], lower healthcare costs, and improve patient satisfaction with their care [18].

Barriers include suboptimal prescribing environments [19], challenges in communicating risks to patients and breakdown of relationships with prescribers [20], and time poverty [21, 22]. Where consultations are suboptimal, misunderstandings can undermine their effectiveness [23]. Computer prompts for physicians, decision-making information and conversation aids [24], training [25] and organisational changes can help support deprescribing efforts [20]. Involving patients in the development of safe deprescribing practices is key to successful implementation [26]. Analyzing the complex interplay of contextual factors affecting implementation success is imperative for interventions to be sustainable [27–31].

Guidance to support the implementation of SMRs states six key requirements [13]: (1) use of appropriate tools to identify qualifying patients; (2) assessment of which patients are most need; (3) formal invitation of patients (supported by carers if necessary) to their SMR; 4. SMRs to be undertaken by trained clinicians; (5) Recording of SMRs on IT systems; and (6) sharing of learning around medicines optimisation between PCNs. The focus of this paper aligns with points 1–4.

To address national requirements, we developed an intervention for deprescribing consultations in general practice to sit within the SMR framework: DEPPLOY (DEprescribing for Problematic PoLypharmacy in Older adults with frailtY), including:

- A validated case-finding tool (AC FRAIL) to identify patients at high risk from medication-related harm, prioritising them for SMR based on the electronic Frailty Index (eFI) [32] and anticholinergic burden [33, 34].
- A process model of patient involvement guided by two stakeholder co-designed documents [35–37]: an Invitation Letter to prepare patients (Additional File 1), and a Stopping Medicines Leaflet to inform patients how/why medicines will be stopped/adjusted (Additional File 2).

### Aim

This study aimed to explore key stakeholders’ perceptions of a co-designed primary care intervention to involve patients and their families in deprescribing and its broader intervention context, guided by the Consolidated Framework for Implementation Research (CIFR).

## Method

### Study design and setting

A qualitative semi-structured interview study was conducted with staff and patients who piloted the DEPPLOY intervention at a primary care site in West Yorkshire, England between April 2022 and January 2023. Interviews were transcribed and anonymized, and data were analysed thematically [38] using a theory-led, a priori approach based on the Consolidated Framework for Implementation Research (CFIR) [27].

### Participants and recruitment

Recruitment was carried out between May 2022 and January 2023. The AC-FRAIL tool [33] was used to identify patients eligible for medication review. An invitation to an SMR consultation was sent to identified patients by post and followed up with a telephone call from staff to explain the invitation letter, answer questions and encourage uptake. A purposive sampling approach recruited patients with frailty taking anticholinergic medications recommended for adjustment or cessation. Staff delivering the DEPPLOY intervention, and senior practice staff responsible for medicines management, were eligible for inclusion in the study. Patients residing in care homes and patients who were terminally ill or lacked capacity to consent were excluded. Participant information sheets were provided to patients and staff and written informed consent taken prior to interview.

### Theoretical approach

This qualitative study took a social constructionist perspective [39], acknowledging that participants recount their experiences in a way that is not objective and unbiased, but reflects their social and cultural milieu and perceptions of the world. CIFR [27, 40] was chosen as an a priori evidence-based framework to enable an exploration of potential barriers and facilitators of implementation in the primary care context.

### Intervention delivery

**Fig. 1:**
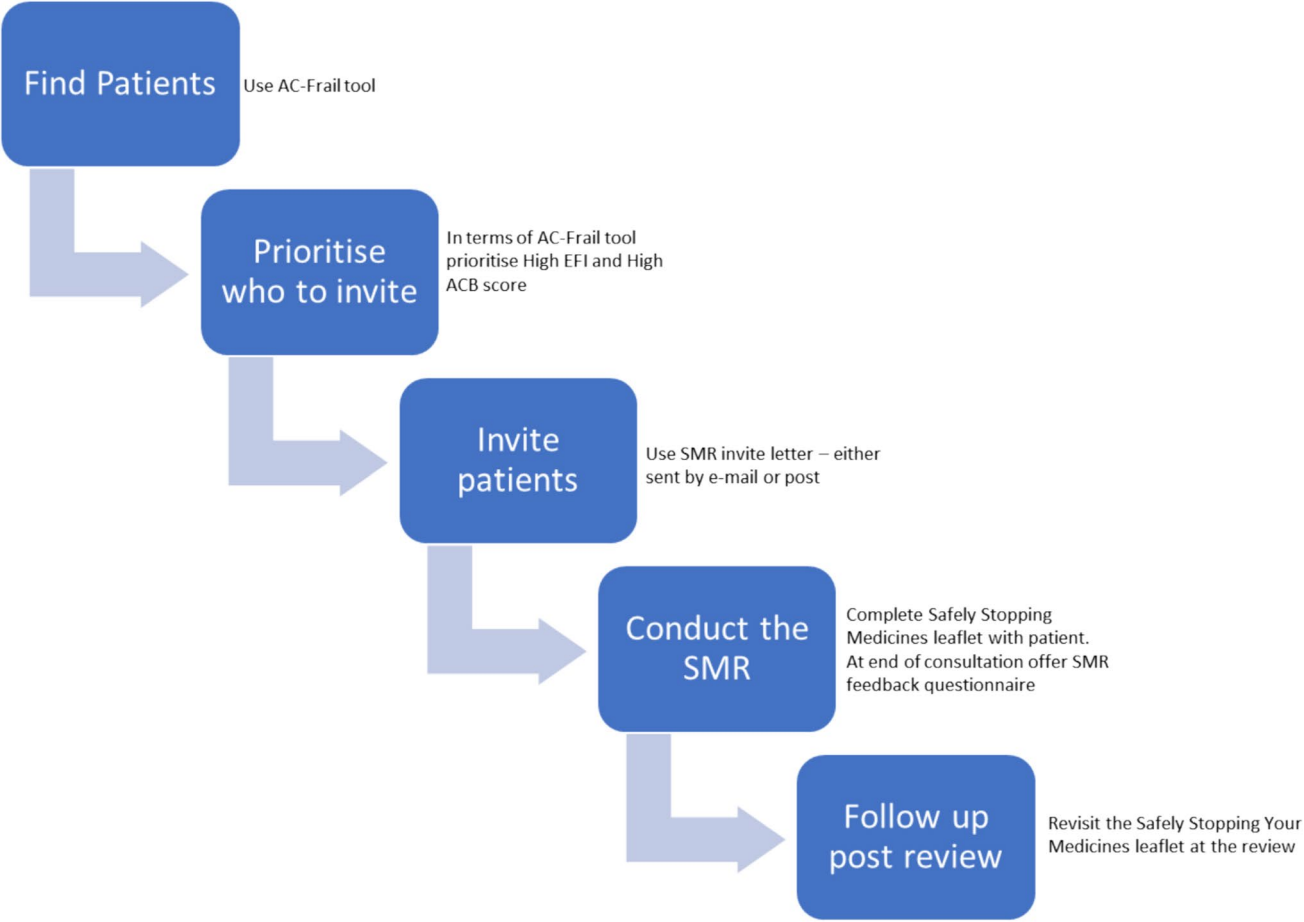
Components of intervention design: mapping the DEPPLOY intervention against the Structured Medicines Review service. *Abbreviations* *AC* Anticholinergic. *ACB* Anticholinergic Burden. *EFI* Electronic Frailty Index. *SMR* Structured Medication Review

The intervention components and delivery of DEPPLOY are shown in Fig. 1. Operationalization was not prescriptive, the aim being to incorporate the components into normal business practice. Practice staff were provided step-by-step guidance on the intervention process. COVID-19 pandemic restrictions coupled with high workloads prohibited face-to-face training. A primary contact within the practice was responsible for disseminating intervention documents.

Pandemic restrictions resulted in the SMRs being conducted by telephone. SMR outcomes were recorded on the Safely Stopping Medicines Leaflet and sent to patients electronically, with details of the SMR consultation.

### Data collection

One-to-one interviews were conducted on the telephone, Teams^R^ or Zoom^R^ by three researchers with 6–20 years of experience in health-related qualitative research (ADO, AD, JB) between May 2022 and January 2023. Interview guides were developed with patient and public involvement, informed by extant literature, piloted, and tailored to reflect participants’ roles and available time (see Additional File 3). All staff involved in the implementation were invited for interview. Staff were interviewed before and after intervention delivery; senior practice staff and patients were interviewed once, after intervention delivery. Interviews were audio recorded using an encrypted recorder, and transcribed verbatim by a university approved provider.

### Data analysis

Data were analysed thematically using a theory-led, a priori approach [38]. Stage 1 of analysis involved familiarisation with data where all transcripts were read repeatedly. In Stage 2, data were coded deductively, using a pre-existing codebook based on the Consolidated Framework for Implementation Research (CFIR) [27]. During this stage, researchers divided transcripts among themselves (ADO, AD, LB), coded them independently, and met regularly to resolve discrepancies in coding through discussion and create new codes which were not captured by the CFIR domains. Stage 3 involved the team reviewing and refining codes until consensus was reached on the coding framework. During Stage 4, LB mapped codes onto CFIR constructs and domains and iteratively adapted the mapping based on input from the wider team. Data were managed in NVivo v12™.

### Patient and public involvement

Patient-facing recruitment materials and interview guides were reviewed by an experienced Patient and Public Involvement representative (NB).

### Ethics approval

Ethical approval was awarded by Wales REC3 (Health and Care Research Wales) Ref:21/WA/0298 17/9/21.

## Results

Five patients and thirteen staff were interviewed (see Table 1), with six staff interviewed twice (24 interviews in total). Interviews lasted between 16 and 59 min; average interview times were 31 min (patients), 40 min (staff), and 27 min (senior staff). All staff had more than 5 years’ experience of deprescribing.

**Table 1:** Participant characteristics

Participants	Number (and gender female (f) and male (m)	Demographics
Patients	5 (4f, 1m)	5 white British
Pharmacists	6 (5m, 1f)	9 white British
General medical practitioners (GPs)	2 (1m, 1f)	2 British Pakistani
Advanced clinical practitioners (ACPs)	2 (m)	1 British Indian
Practice manager	1 (f)	1 British (other)
Administrative staff	2 (f)	

Analysis is presented using constructs from the Consolidated Framework for Implementation Research [27] (see Table 2 for domains, constructs and additional quotes). This a priori theoretical lens demonstrated the barriers and facilitators of embedding the intervention in a primary care setting, and the trade-offs between the two, situated within the broader context of implementation.

**Table 2:** CFIR domains and constructs identified in the interviews [27]

Domain	Construct	Short description	Examples of additional quotes supporting constructs
Innovation	A. Innovation Source	The group that developed and/or visibly sponsored use of the innovation is reputable, credible, and/or trustable	**Innovation Evidence Base** Patients were receptive to discontinuing medication: the SMR Invite Letter encouraged questions and enhanced understanding of the benefits of deprescribing: *It’s that understanding. It reduces noise. The patients have got an understanding why they are on that [medication].* (Practice Manager, Senior staff interview 11) **Relative Advantage** Awareness of current deprescribing tools (outside of the DEPPLOY intervention) were limited and not always incorporated into standard practice by staff *I mean, there are thousands of these tools and I never know which one is up to date…when it comes to deprescribing, it's holistic. And does a template help with holistic care and is the template actually ticking the boxes that need to be ticked?* (GP, Staff interview 3)
	B. Innovation Evidence Base	The innovation has robust evidence supporting its effectiveness	
	C. Innovation Relative Advantage	The innovation is better than other available innovations or current practice	
	D. Innovation Adaptability	The innovation can be modified, tailored, or refined to fit local context or needs	
	E. Innovation Trialability	The innovation can be tested or piloted on a small scale and undone	
	F. Innovation Complexity	The innovation is complicated, which may be reflected by its scope and/or the nature and number of connections and steps	
	G. Innovation Design	The innovation is well designed and packaged, including how it is assembled, bundled, and presented	
	H. Innovation Cost	The innovation purchase and operating costs are affordable	
Outer setting	A. Critical Incidents	Large-scale and/or unanticipated events disrupt implementation and/or delivery of the innovation	**Local attitudes** However it was also perceived that as the practice was research-active staff might see this as yet another initiative to test and have a lack of deeper knowledge of the intervention and impact *I think sometimes there’s an element of, oh, this is just going to be another piece of work or something else that we need to think about… So, there might be just that element of, I suppose, somehow evolving that understanding of the wider organisation around what this project is and the benefits of it.* (ACP, Senior Staff interview 12)
	B. Local Attitudes	Sociocultural values (e.g., shared responsibility in helping recipients) and beliefs (e.g., convictions about the worthiness of recipients) encourage the Outer Setting to support implementation and/or delivery of the innovation	
	G. External Pressure	External pressures drive implementation and/or delivery of the innovation	
Inner setting	A. Structural Characteristics	Infrastructure components support functional performance of the Inner Setting	**Information Technology Infrastructure** SMRs were delivered remotely via telephone as per usual clinical practice. This was convenient for both the pharmacist and patient and considered safe. Whilst operationally effective for pharmacists, it had mixed reviews from patients *Over the phone, I really don’t think you can have a discussion, because things don’t crop up…It’s just ‘This, this, this, this’, whereas if it’s face to face, you can have a discussion about things, I think, and it can be made plainer, or make you understand more.* (Patient 3) **Access to Knowledge & Information** The practice manager, who was supporting staff delivering the intervention, reported that the training to contribute to the intervention delivery was effective and no additional training was required: *Our head pharmacist, is good at communicating…They explained thoroughly what it was all about, what we needed to… Where we needed to be and what we needed to be. So, no, absolutely fine.* (Practice Manager, Senior Staff interview 11)
	2. Information Technology Infrastructure	Technological systems for tele-communication, electronic documentation, and data storage, management, reporting, and analysis support functional performance of the Inner Setting	
	F. Compatibility	The innovation fits with workflows, systems, and processes	
	I. Mission Alignment	Implementing and delivering the innovation is in line with the overarching commitment, purpose, or goals in the Inner Setting	
	K. Access to Knowledge & Information	Guidance and/or training is accessible to implement and deliver the innovation	
Individuals	E. Implementation Leads	Individuals who lead efforts to implement the innovation	**Implementation Team Members** To make this intervention more sustainable key parties would need to be involved: *Well, all of the prescribers, really. I know that’s quite general, but what you don’t want is your prescribers undoing the work that you’ve done.* (ACP, Senior Staff interview 13)
	F. Implementation Team Members	Individuals who collaborate with and support the Implementation Leads to implement the innovation, ideally including Innovation Deliverers and Recipients	
	Characteristic subdomain		
	A. Need	The individual(s) has deficits related to survival, well-being, or personal fulfillment, which will be addressed by implementation and/or delivery of the innovation	Another view was that a combination of key roles would be highly effective in leading this intervention if embedded as usual practice: *Although we work as a team, components of the team will have their different clinical leads. So, I think, yes, certainly our clinical lead pharmacist. Somebody like myself with the strategic role, and then also we have one of our GPs with the special interest in diabetes as well. So, I think between the three of us, across all the different levels of the clinicians that would implement a tool such as this*. (ACP, Senior Staff interview 12)
	B. Capability	The individual(s) has interpersonal competence, knowledge, and skills to fulfill Role	
	D. Motivation	The individual(s) is committed to fulfilling Role	
Implementation process	A. Teaming	Join together, intentionally coordinating and collaborating on interdependent tasks, to implement the innovation	No additional quotes available
	H. Reflecting & Evaluating	Collect and discuss quantitative and qualitative information about the success of implementation and/or the innovation	
	I. Adapting	Modify the innovation and/or the Inner Setting for optimal fit and integration into work processes	

### Domain—innovation

#### Source

The intervention was co-designed involving patients, carers and healthcare professionals, focused on primary care settings [8, 33, 36, 37]. This was in response to a need to improve patient satisfaction with deprescribing consultations as part of the wider SMR provision, the appetite to better support patients through deprescribing, and government focus on appropriate prescribing.

### Evidence base

The findings indicated the intervention facilitated greater patient involvement in the SMR process, from preparation to follow-up, which enhanced patient satisfaction:

*80% of patients verbally sort of showed they were happy with the review…it wasn’t anything to do with what I did, I guess, it was the entire thing. It was just they felt really like listened to…it felt like there was a lot more willingness for that conversation to happen.* (Pharmacist, staff interview 4).

#### Relative advantage

The intervention had multiple components. The use of the AC-FRAIL tool [29] enabled systematic identification of patients most in need of deprescribing (those with moderate-severe frailty and a medium–high ACB, using high-risk medicines). Targeting patients most in need using AC-FRAIL made the DEPPLOY process manageable and relevant to staff, and SMR consultations increased in focus:

I think it's a great idea to invite people based on a frailty index. I think the only way to really deprescribe and do it really properly rather than tiny wins of one medication is to ask the patient, which tablets do you not want to take anymore? It has to be patient-led. (GP, Staff interview 03).

#### Adaptability

Staff were positive about the introduction of the intervention into their existing service provision:

Really well, yes…We’re constantly trying to create pathways to make sure we deliver better care to as many patients as we possibly can. So, this sounds exactly like it would be all part of what we would want to do. (ACP, Senior Staff interview 13).

#### Trialability

The practice opted to initiate SMR clinics outside of usual practice, allowing the intervention to be extended/removed without patient harm or operational damage. The clinics were operated on a weekly basis and patients were identified and booked into slots for consultations by the care coordinator followed by the explanatory phonecall:

I just thought, rather than upsetting patients by thinking, well, they sent me a letter, but nobody’s rang me, what’s that about? And that’s something else for them to complain about. (Care Coordinator, Staff interview 08).

#### Complexity

The intervention had 4 main components (plus a follow up consultation), each with individual purpose and contribution to patient care. Operationally, the implementation of the DEPPLOY components in the practice required administrative support, adding time and complexity into existing workflow.

I don’t know what the answers are, but when we were attaching all the paperwork and filling out the Stopping Medicines Safely thing…all that was a little bit bitty and a little bit faffy…It wasn’t smooth…that was a little bit discouraging. (Pharmacist, staff interview 9).

#### Design

The DEPPLOY intervention components were perceived as logical by staff, with a clear function and benefit to the practice and its patients in supporting the planning and delivery of SMRs. The AC-FRAIL tool had clear utility in identifying at-risk patients:

It’s the only way to actually do it, because otherwise the way I guess we were doing it before, it’s very easy to almost miss the anticholinergic burden, when you’re dealing with all the other medical issues. … A lot of very high-risk medicines that had been identified, so whichever tool it was that they’ve used to pick up on these, it was impressive. (Pharmacist, staff Interview 7).

#### Cost

Staff acknowledged the additional time needed to complete the SMR consultations:

In a DEPPLOY [SMR]…you have more time to then discuss reducing medications, whereas in real life, you might have only two, three minutes to look at the deprescribing side of things… I think the deprescribing becomes more my priority when I know I’m doing it as a part of DEPPLOY SMR. (Pharmacist, staff interview 6).

#### Domain—outer setting

##### Critical incidents

Introducing this intervention (DEPPLOY) was delayed and complicated due to the COVID-19 pandemic. Participants noted the value of additional training to support its delivery; the pandemic impacted on the ability to provide this during the pilot.

If I’m honest, during the reviews, I didn’t notice a huge difference in how patients were prepared, but I don’t think… If I’m honest, because of the limited training we’d had with it, I wasn’t fully aware that the patient had actually been sent all this. (Pharmacist, staff interview 7).

#### Local attitudes

The perception was that the delivery of SMRs was good practice and patients benefitted from this activity:

The only way to do it [SMRs] is target it, which is what you’re doing in a targeted and supportive way for the patient, which again leads to big changes for patients that they’d have a lot of worries about…. (ACP, Senior staff interview 13).

#### External pressure

There was external pressure to undertake SMRs and this was target influenced. The adoption of DEPPLOY was considered to align with the need to complete medication reviews to meet targets and secure income.

…. it’s like a lot of these targets. It’s related to payment, so if you don’t, if you choose not to do it, you won’t get paid, but that’s the way a lot of these targets work, is you get income to the practice related to achievement for certain targets. …. (GP, Senior Staff interview 10).

#### Domain—inner setting

##### Structural characteristics

The host site was part of a larger practice with experience of delivering SMRs in primary care and care home settings, and dedicated time and staff resources allocated to testing DEPPLOY components.

We’re very lucky here in that we have a good number of pharmacists who are employed, and other clinicians who would be interested in this, but it’s, I suppose, getting the most out of the resources that are available. (GP, Senior Staff interview 10).

##### Information technology infrastructure

The delivery of the intervention was via telephone utilising standard clinical, management and reporting practices. Care coordinators conducted phonecalls with patients to support appointment bookings.

So, that’s why these care coordinators were brought in, probably more so me, because I now ring, whereas the others, they send the links…when I notice the gaps, because they’re not responding to the letters, they’re not responding to the links. I have the time to ring them. (Care Coordinator, Staff interview 08).

#### Compatibility

Staff reflection on the intervention was positive and felt that it could be integrated into current practice:

I think it’s [DEPPLOY] very compatible. I think it’s a good way of looking at doing the medication review…. I like the invite letter. The feedback that’s given to the patients on the Safely Stopping Medicines looks, it fits in nicely with what we want to do with regards to stopping medications, and, well, making medications more safe and effective. (GP, Senior Staff interview 10).

##### Mission alignment

Engagement with medication reviews also helped support other internal objectives such as enhanced inter-professional activity. Staff felt that they would benefit from more training in this area to maximize the impact of medication reviews:

Like, we could, a bit of training, bit more training, and also just having more of a way of working better between teams. So, having that MDT-style team, where you’ve got the input of a nurse, GP, pharmacist, maybe working that better, so you’ve got that support and got different viewpoints while you’re doing the reviews. (Pharmacist, Staff interview 7).

##### Access to knowledge and information

Staff advised that when additional information/training was required this was provided by a colleague to prepare them for the delivery of the intervention. However, some pharmacists delivering the intervention felt that the training was lacking and that this left them unprepared.

I had a chat, like, a pretty informal chat with [de-identified] in regards to DEPPLOY, and she just explained what the research was around, and just seeing if there’s a way we could facilitate SMRs better. There wasn’t particularly any training on how to do the actual consultation. (Pharmacist, Staff interview 6).

#### Domain—Individuals

##### Implementation leads

Whilst a broad team of contributors, there was a strong feeling that medication reviews in primary care should be led by pharmacists to ensure effective implementation:

A community pharmacist would know what medications a patient’s on, but wouldn’t have all the chronic patient record, where in primary care, our pharmacists that we directly employ can see the full medical record, can see all the medications, and can liaise with other clinicians for advice if needed. So, I think it is ideal to be done in primary care…I think a fully trained pharmacist would do this very, very well. (GP, Senior Staff interview 10).

##### Implementation team members

There were many practice staff involved in the planning and delivery of the intervention. The inclusion of multiple healthcare professionals in this process was considered important.

I think it’s so important that we embrace and utilise all the skills that different healthcare professionals bring, ….when it comes to the medication reviews and the reviews around some of these chronic diseases, with the right support and training, various healthcare professionals are more than capable of conducting these reviews. (ACP, Senior Staff interview 12).

##### Need

Delivering SMRs is part of the pharmacist/GP role and this intervention supported and enhanced their personal fulfilment in supporting their patients.

…… for myself it was really positive, I felt like the reviews ran so much more smoothly. And I’ve got the outcome that we, both the patient and myself are happy with at the end of it, so I have no issues whatsoever with the process. And even the follow ups afterwards were just as smooth. (Pharmacist, Staff Interview 4).

##### Capability

The practice had a high level of research readiness and human resources to support the intervention pilot. Pharmacists, based on their expertise and interpersonal skills, were considered the most appropriate healthcare professional group to lead this intervention:

I think the pharmacists, because obviously they’ve got that knowledge, and they’re very good at meeting up with us and explaining things very clearly, so you know what’s happening. (Practice Manager, Senior Staff interview 11).

##### Motivation

Staff were supportive of the use of DEPPLOY to support deprescribing, and patients developed an understanding of deprescribing, and of the purpose of the intervention and its associated benefits.

I think I’d see more deprescribing taking place. I felt like, when I saw the word DEPPLOY, and I was going into the review to do it, it affects your mindset. I think most of the time, I go in, and I know I’m going to try to de-prescribe, but the emphasis I put on deprescribing might not have been the same in another review where it didn’t say DEPPLOY. (Pharmacist, staff interview 6).

#### Domain – Implementation Process

##### Teaming

The DEPPLOY intervention involved the coordination of activity by multiple roles within the practice team including senior leaders, administrators and pharmacists. A team of support staff worked to set up the medicines reviews appointments to ensure delivery of SMRs.

His [lead pharmacist] pharmacy team do the medication reviews and BP reviews and stuff, so I’ve got reports that I print off for patients. So, I get everybody booked in for medication reviews (Care Coordinator, Staff interview 08).

##### Reflecting and evaluating

Regular feedback was maintained throughout the intervention testing to ensure delivery and feedback was provided by staff delivering SMRs via interview as part of the study. Patient experience of the value of SMRs and the process was also captured:

I think it’s a brilliant idea. I know people that have been taking medication for years and they haven’t a clue what they’re taking or why they’re taking it, and I think that’s dangerous. (Patient 2).

##### Adapting

The practice implementation team introduced an additional step into the intervention, a phonecall to support the medication review invitation letter, which was not planned but thought necessary to avoid patient confusion and non-attendance.

So, I would ring them and say, I’ll pop you a letter in the post. It will explain it all, and then I just highlighted their time,…. and if you have any questions and that, so yes. (Care Coordinator, Staff interview 08).

## Discussion

### Statement of key findings

This study explored the introduction of an intervention (DEPPLOY) in a primary care setting, to gain insight into the contextual factors that impacted its implementation. The CFIR mapping (see Table 2) highlighted a strong focus on the innovation domain. Fewer views were reported relating to the other domains, although relevant insights were captured.

The DEPPLOY components were co-designed with the view that they could enhance the operationalization of the deprescribing process within SMR consultations, increase patient engagement in the process and ensure patient safety and enhanced patient satisfaction. The intervention had a robust and credible evidence base to support this research study [8, 33, 35–37] (Innovation domain).

The findings of this study indicated that the DEPPLOY process components were perceived as beneficial to deprescribing within the SMR process, with some caveats. Pharmacists could see the benefit of using AC-FRAIL to proactively identify patients for targeted deprescribing discussions. The DEPPLOY intervention increased understanding and investment in deprescribing among patients and provided individualized written documentation focused on reducing medications. Burdens included increased preparation and delivery time for SMRs, which lasted approximately 30 min plus 15 min administration time (Innovation domain). Lack of experience of SMRs among some staff impacted on their understanding of the intervention (Inner Setting domain). Successful integration will require further attention to the intervention’s ‘fit’ within existing systems (Implementation Process domain) which is desirable if payment follows SMR completion (Outer Setting domain). The benefits and burdens associated with its operationalization were, however, seen as trade-offs. In particular, the additional time invested in the DEPPLOY SMR increased patient understanding of and investment in deprescribing, improved review communication and outcomes, potentially saving medication costs, and reduced the need for follow-up. It also led to greater integration of the pharmacist’s role and staff were keen to extend interprofessional learning opportunities to build on this to maximize the potential of medicines reviews (Inner Setting domain).

Pharmacists were seen as clear leads of SMRs based on their expertise and role within the clinical team (Individuals Setting). SMRs reflected their extended role as healthcare professionals but also were seen as the perfect opportunity to boost interprofessional learning and team dynamics. The facilitation of the intervention was very much seen as a team effort with clear role demarcation within a larger practice team, working together to deliver the DEPPLOY components.

### Interpretation – Barriers to implementation success

The key barriers to implementation reported were time; complexity of intervention; remote delivery and lack of training.

Time pressures are a known barrier in deprescribing consultations [21, 22]. However, NHS England guidance stipulates that an SMR should take longer than the average GP appointment, 30 min or more, with the length based on case complexity [13]. Extra time was factored into the delivery of the SMRs in dedicated clinics as part of this intervention. The DEPPLOY implementation demonstrates that careful planning is required to integrate longer SMRs into healthcare practitioners’ operational caseload, and the need for administrative resources to support wider delivery. A time and motion review of activity may lead to reduction of time for the overall process.

Optimal testing and customizing of the intervention to the inner setting added extra steps into the SMR process. This required commitment of additional resources and time. As the host site was well-established, summoning resources was feasible, but this could be problematic for smaller settings with less resources to instigate organizational changes needed to support deprescribing activities [20]. Smaller practice sites may opt to test individual components of the intervention to match their resources.

Lack of training and remote delivery of the SMRs by telephone also yielded negative responses. Staff directly delivering the intervention felt unprepared to deliver the consultation effectively due to inadequate training [25]. This conflicts with expectations of SMR delivery by NHS England [13]. Inclusion of SMR training can be factored into professional development upskilling (practice or professional stipulation). Patients also felt that whilst their consultations were sufficient, some would have preferred in-person consultations to be more involved in the deprescribing process [26]. Practices could therefore consider a hybrid (remote and in-person) approach to SMR delivery.

### Interpretation – Facilitators of implementation success

The key facilitators of implementation reported were the DEPPLOY components; organizational readiness; intervention-organisational fit; patient engagement; intervention and financial incentives.

Use of the AC-FRAIL tool to identify patients who would benefit from deprescribing was seen as a positive and manageable first step. Organisational readiness to support deprescribing innovations is a key precursor to success [19, 20]. The pilot site had a high level of research readiness and resource richness so could capably handle testing this intervention. The intervention could be sympathetically integrated into a practice in a non-prescriptive manner: the host site introduced adaptations that were operationally effective. For example, staff reported that patients did not always read corporate communications, so the decision was made to contact patients by telephone prior to receiving the SMR invitation to avoid confusion.

Patients benefit from guided preparation for deprescribing consultations [35, 37] – timely, targeted information can develop their understanding of the benefits of SMRs. The DEPPLOY intervention facilitates this understanding, and additionally, has the potential to reduce risks around the deprescribing process, such as concerns about medication changes and a breakdown in trust in the patient/professional relationship [20]. The intervention supported patient empowerment and engagement not only in the preparation phase, but provided a record of their SMR consultation, and medicines that were stopped. This increased patients’ confidence (a key driver of engagement in SMRs [41]) and replaced burdensome personal mechanisms to support medicines management – for example, patients writing down what they remember from the consultation, which may be subject to errors in recall or accuracy.

The DEPPLOY intervention was co-designed drawing on patient and clinician lived/working experience. It is novel in purpose and format, and proved successful in responding to NHS England policy expectations, whilst existing tools were lacking in adoption. In-depth analysis using CIFR demonstrated whilst the intervention is sympathetic to current operational activity, to be sustainable it needs to be responsive to changing requirements. Patients wanted more in-person face-to-face consultations, whereas the SMR consultations were conducted by telephone (also captured as a barrier). Since completion of the intervention two of the DEPPLOY intervention components (Invitation letter and Safely Stopping Medicines leaflet) have been adopted more widely to support SMRs and deprescribing consultations within the UK [42].

The completion of SMRs was reported as a driver for this activity (Outer Setting domain). Payments are released for this activity as part of the Primary Care Network Directed Enhanced Service (PCN DES).

### Strengths and limitations

Despite conducting this study during the COVID pandemic, a rich data set including insights from patient and staff experiences of the DEPPLOY intervention was developed, supplemented by the application of CIFR [27] to explore contextual issues around the operational impact of the implementation within a primary care setting. However, the pandemic impacted on recruitment and data collection and made on-site training unfeasible. Training was flagged as a necessity for future implementation. Limitations of the study also included single site analysis and the small patient sample size. We addressed potential issues of transferability across contexts using CIFR and acknowledge that the patient sample was homogeneous and did not capture the diversity of the potential population for this intervention. The application of CFIR was retrospective and we did not aim to utilize the full extent of this framework analysis. This is deemed acceptable as a research strategy by the framework authors [40]. We did not aim to evaluate the effectiveness of the DEPPLOY intervention, which would require a pilot randomised controlled trial (RCT), followed by a definitive RCT.

## Conclusion

The study findings indicate that patients and staff understood the value of the DEPPLOY deprescribing intervention. The intervention has the potential to support deprescribing in a way that realizes operational and patient benefits but also embraces the extension of the pharmacist’s role within clinical teams. The AC-FRAIL tool was an effective mechanism to search for patients with moderate to severe frailty and high ACB to invite for an SMR consultation. This was a novel resource which did not exist in the practice. Staff were confident that the search outputs were accurate and acted on these. Patients benefited from the information shared in the SMR Invitation Letter and the record of decisions and medication changes in the Safely Stopping Medicines Leaflet, feeling more prepared and empowered in the SMR process. The DEPPLOY intervention can be adopted within primary care settings as a complete package, or in individual components, to support patient-centered medication consultations. For future use, the Safely Stopping Medicines Leaflet may need to be extended to address other issues pertinent to SMR follow-up, such as starting a new medicine or reducing a dose. Key barriers and facilitators to implementation were identified that should be considered by other researchers aiming to test like interventions in primary care settings. The learnings from this analysis have been applied to the next step of implementation within care homes (DEPPLOY-CH).

## Supplementary Information

Below is the link to the electronic supplementary material.

Supplementary file1: Additional File 1 Patient Invitation Letter. (DOCX 1071 KB)

Supplementary file2: Additional File 2 Stopping Medicines Leaflet. (DOCX 26 KB)

Supplementary file3: Additional File 3 Interview Guides. (DOCX 40 KB)

Supplementary file4: Additional File 4 COREQ checklist. (DOCX 32 KB)

## Data Availability

Data generated during this study are available via the corresponding author on request.
