# Correction: A consolidated framework for implementation research (CFIR) informed exploration of a primary care intervention to support deprescribing for problematic polypharmacy in older adults living with frailty (DEPPLOY) in England: a qualitative study

**DOI:** 10.1007/s11096-026-02169-1

**Published:** 2026-06-08

**Authors:** Liz Breen, Amrit Daffu-O’Reilly, Aliya Darr, Jonathan Benn, Beth Fylan, Jonathan Silcock, George Peat, D. K. Theo Raynor, Duncan R. Petty, Syed Tabish Zaidi, Alison Bravington, Nazreen Butt, Hannah Hartley, David P. Alldred

**Affiliations:** 1 University of Bradford, Bradford, UK; 2 Keele University, Newcastle, UK; 3 University of Leeds, Leeds, UK; 4 NIHR Yorkshire and Humber Patient Safety Research Collaboration, Bradford Teaching Hospitals NHS Foundation Trust, Bradford, UK; 5 University of Northumbria, Newcastle, UK; 6 University of Tasmania, Hobart, Australia

**Correction to: International Journal of Clinical Pharmacy** 10.1007/s11096-026-02140-0

The author name Daisy Halligan has now been removed from the author group. The correct author group is presented in this erratum.

Additionally, in Table 2, the “Construct” and “Short description” columns were misaligned. Please find the corrected version of Table 2 provided below. The original article has been corrected.

**Table 2:** CFIR domains and constructs identified in the interviews [27]

Domain	Construct	Short description	Examples of additional quotes supporting constructs
Innovation	A. Innovation Source	The group that developed and/or visibly sponsored use of the innovation is reputable, credible, and/or trustable	**Innovation Evidence Base** Patients were receptive to discontinuing medication: the Safely Stopping Medicines Leaflet encouraged questions and enhanced understanding of the benefits of deprescribing: *It’s that understanding. It reduces noise. The patients have got an understanding why they are on that [medication].* (Practice Manager, Senior staff interview 11)
	B. Innovation Evidence Base	The innovation has robust evidence supporting its effectiveness	
	C. Innovation Relative Advantage	The innovation is better than other available innovations or current practice	
	D. Innovation Adaptability	The innovation can be modified, tailored, or refined to fit local context or needs	
	E. Innovation Trialability	The innovation can be tested or piloted on a small scale and undone	
	F. Innovation Complexity	The innovation is complicated, which may be reflected by its scope and/or the nature and number of connections and steps	**Relative Advantage** Awareness of current deprescribing tools (outside of the DEPPLOY intervention) were limited and not always incorporated into standard practice by staff. *I mean, there are thousands of these tools and I never know which one is up to date...when it comes to deprescribing, it's holistic. And does a template help with holistic care and is the template actually ticking the boxes that need to be ticked?* (GP, Staff interview 3)
	G. Innovation Design	The innovation is well designed and packaged, including how it is assembled, bundled, and presented	
	H. Innovation Cost	The innovation purchase and operating costs are affordable	
Outer setting	A. Critical Incidents	Large-scale and/or unanticipated events disrupt implementation and/or delivery of the innovation	**Local attitudes** However it was also perceived that as the practice was research-active staff might see this as yet another initiative to test and have a lack of deeper knowledge of the intervention and impact. *I think sometimes there’s an element of, oh, this is just going to be another piece of work or something else that we need to think about... So, there might be just that element of, I suppose, somehow evolving that understanding of the wider organisation around what this project is and the benefits of it.* (ACP, Senior Staff interview 12)
	B. Local Attitudes	Sociocultural values (e.g., shared responsibility in helping recipients) and beliefs (e.g., convictions about the worthiness of recipients) encourage the Outer Setting to support implementation and/or delivery of the innovation	
	G. External Pressure	External pressures drive implementation and/or delivery of the innovation	
Inner setting	A. Structural Characteristics	Infrastructure components support functional performance of the Inner Setting	**Information Technology Infrastructure** SMRs were delivered remotely via telephone as per usual clinical practice. This was convenient for both the pharmacist and patient and considered safe. Whilst operationally effective for pharmacists, it had mixed reviews from patients. *Over the phone, I really don’t think you can have a discussion, because things don’t crop up…It’s just ‘This, this, this, this’, whereas if it’s face to face, you can have a discussion about things, I think, and it can be made plainer, or make you understand more.* (Patient 3) **Access to Knowledge & Information** The practice manager, who was supporting staff delivering the intervention, reported that the training to contribute to the intervention delivery was effective and no additional training was required: *Our head pharmacist, is good at communicating...They explained thoroughly what it was all about, what we needed to… Where we needed to be and what we needed to be. So, no, absolutely fine.* (Practice Manager, Senior Staff interview 11)
	2. Information Technology Infrastructure	Technological systems for tele-communication, electronic documentation, and data storage, management, reporting, and analysis support functional performance of the Inner Setting	
	F. Compatibility	The innovation fits with workflows, systems, and processes	
	I. Mission Alignment	Implementing and delivering the innovation is in line with the overarching commitment, purpose, or goals in the Inner Setting	
	K. Access to Knowledge & Information	Guidance and/or training is accessible to implement and deliver the innovation	
Individuals	E. Implementation Leads	Individuals who lead efforts to implement the innovation	**Implementation Team Members** To make this intervention more sustainable key parties would need to be involved: *Well, all of the prescribers, really. I know that’s quite general, but what you don’t want is your prescribers undoing the work that you’ve done.* (ACP, Senior Staff interview 13)
	F. Implementation Team Members	Individuals who collaborate with and support the Implementation Leads to implement the innovation, ideally including Innovation Deliverers and Recipients	
	Characteristic subdomain		
	A. Need	The individual(s) has deficits related to survival, well-being, or personal fulfillment, which will be addressed by implementation and/or delivery of the innovation	Another view was that a combination of key roles would be highly effective in leading this intervention if embedded as usual practice: *Although we work as a team, components of the team will have their different clinical leads. So, I think, yes, certainly our clinical lead pharmacist. Somebody like myself with the strategic role, and then also we have one of our GPs with the special interest in diabetes as well. So, I think between the three of us, across all the different levels of the clinicians that would implement a tool such as this*. (ACP, Senior Staff interview 12)
	B. Capability	The individual(s) has interpersonal competence, knowledge, and skills to fulfill Role	
	D. Motivation	The individual(s) is committed to fulfilling Role	
Implementation process	A. Teaming	Join together, intentionally coordinating and collaborating on interdependent tasks, to implement the innovation	No additional quotes available
	H. Reflecting & Evaluating	Collect and discuss quantitative and qualitative information about the success of implementation and/or the innovation	
	I. Adapting	Modify the innovation and/or the Inner Setting for optimal fit and integration into work processes	

