# Supplementary material for: A consolidated framework for implementation research (CFIR) informed exploration of a primary care intervention to support deprescribing for problematic polypharmacy in older adults living with frailty (DEPPLOY) in England: a qualitative study
Source: Int J Clin Pharm. 2026 Apr 27;48(4):1594–606. doi: 10.1007/s11096-026-02140-0 (PMC13368935; doi:10.1007/s11096-026-02140-0)
Supplement: Supplementary file 1 — Supplementary file1: Additional File 1 Patient Invitation Letter. (DOCX 1071 KB) [file 11096_2026_2140_MOESM1_ESM.docx]

Anytown Practice

High Street

Anytown

Dear

**Reviewing your medicines**

The practice is running a new service to help you with your medicines - this is called a:

- Structured Medicines Review.

A medicine review is a chance to check that your medicines are the best ones for you.

**What will happen at the review?**

- You will have a telephone call with the practice pharmacist.
- They will check your medicines are working - and not causing side effects.
- It is also a chance for you to tell us how you are getting on with your medicines - and to ask questions and find out more about them.

**What happens next?**

We will contact you to make an appointment to speak with the practice pharmacist over the telephone.

- The pharmacist will explain what your medicines are for.
- They will check if any changes to your medicines are needed.
- There will also be a chance to have your questions answered.

If someone helps you with your medicines, it may be helpful for them to be with you when you speak to the practice pharmacist.

**Why are we doing this?**

We are doing this to make sure that your medicines are the right ones for you.

- The purpose of the review is not to save money.
- Also it is not to check if you are taking your medicines.

No medicines will be altered without agreement between you and the pharmacist or GP.

On the other side there are some questions you might want to ask about your medicines at the appointment.

Yours sincerely

Dr

**Questions to think about before the appointment**

- How do my medicines work?
- How do I know they are helping me?
- Do I need to take them still?
- Why do I have to take so many pills?
- What side effects do they cause?
- It is difficult for me to open the containers - can you help with this?
- It is difficult to remember to take my medicines – can you help with this?
- I run out my medicines at different times – can you make this the same time for all of them?
