# Supplementary material for: A consolidated framework for implementation research (CFIR) informed exploration of a primary care intervention to support deprescribing for problematic polypharmacy in older adults living with frailty (DEPPLOY) in England: a qualitative study
Source: Int J Clin Pharm. 2026 Apr 27;48(4):1594–606. doi: 10.1007/s11096-026-02140-0 (PMC13368935; doi:10.1007/s11096-026-02140-0)
Supplement: Supplementary file 2 — Supplementary file2: Additional File 2 Stopping Medicines Leaflet. (DOCX 26 KB) [file 11096_2026_2140_MOESM2_ESM.docx]

**Safely stopping your medicine**

Today we have agreed that you should stop your medicine: _______________________

- This side of the leaflet outlines what to do when stopping this medicine
- The other side has information if you need to stop gradually.

Keep a copy of this with you – and show it to anyone you see about your health.

**Why we have agreed to stop this medicine**

Medicines should only be used when they benefit you. In your case, we have agreed the benefits of this medicine are less than the risk of side effects caused by it.

**How will I stop this medicine?**

Stop taking your medicine straight away from: ______________

Stop taking your medicine gradually - see the other side of this leaflet

**What might I notice and what should I do?**

Most people do not notice any difference after stopping. But do keep watching for any changes in your condition.

**Minor symptoms**

- Continue as agreed and keep watching for any changes in your condition.
- Talk to your practice pharmacist about these symptoms at your next appointment

**Major symptoms**

- Call your practice straight away for help or call 111 if after hours
- Severe symptoms - call 999 for help

**What should I do next?**

Your next appointment is: ……………………………………….

If you need to speak to somebody before ring …………………………………….

and ask for: ……………………………………….

**If you are gradually stopping your medicine**:

………………………………………………………………………………………………………………………………………………………………………………………………………………………………………………………………………………………………………………………………………………………………………………………………………………………………………………………………………………………………………………………………………………………………………………………………………………………………………………………………………………………………………………………………………………………………………………………………………………………………………………………………………………………………………………………………………………………………………………………………………………………………………………………………………………………………………………………………………………………………………………………………………………………………………………………………………………………………………………………………………………………………………………………………………………………………………………………………………………………………

**Additional information**

………………………………………………………………………………………………………………………………………………………………………………………………………………………………………………………………………………………………………………………………………………………………………………………………………………………………………………………………………………………………………………………………………………………………………………………………………………………………………………………………………………………………………………………………………………………………………………………………………………………………………………………………………………………………………………………………………………………………………………………………………………………………………………………………………………………………………………………………………………………………………………………………………………………………………………………………………………………………………………………………
