# Supplementary material for: A consolidated framework for implementation research (CFIR) informed exploration of a primary care intervention to support deprescribing for problematic polypharmacy in older adults living with frailty (DEPPLOY) in England: a qualitative study
Source: Int J Clin Pharm. 2026 Apr 27;48(4):1594–606. doi: 10.1007/s11096-026-02140-0 (PMC13368935; doi:10.1007/s11096-026-02140-0)
Supplement: Supplementary file 3 — Supplementary file3: Additional File 3 Interview Guides. (DOCX 40 KB) [file 11096_2026_2140_MOESM3_ESM.docx]

**Interview Guide: Patients who agreed to deprescribe, v1.4**

| **PREPARATION** | |
| --- | --- |
| In advance of the interview:   - Ask the participant if they could bring their ‘safely stopping your medicines’ leaflet to the interview. - Clarify that patient can be interviewed with the support of a family member, friend, or carer, if they wish. | |
| **INTRODUCTION** | |
| - Introduce the interview, explain purpose and clarify participant’s understanding of purpose of interview. - Confirm that the participant is happy for the interview to take place, and for the interview to be audio recorded. - Reassure the participant that they can take a break during the interview, and that it is fine if they do not want to answer any of the questions. - Reiterate to the participant that the purpose of the interview is not to make any decisions about their medicines or care, but to explore their views on reducing or stopping medicines and their experiences of recently reducing the dosage of a medicine or stopping one or more medicines. - Take consent. | |
| **Questions** | **Prompts** |
| **OPENING QUESTION** | |
| How do you feel about the medicines you take? (In general, as opposed to focused on medicines deprescribed.) | How important are your medicines to you? (and your health)  Do you know the purpose of each of your medicines/what they do?  Do any of these words apply to your thoughts about your own medicines?: content or not content with them/happy or not happy/ frustrated or satisfied?  And what about your medicines in general: content or not content with them/happy or not happy/ frustrated or satisfied? |
| **PREVIOUS EXPERIENCES** | |
| Before you had this appointment had you ever felt like stopping or reducing one or more of your medicines? Can you explain why? | What made you feel that way? |
| **EVALUATING DEPPLOY** | |
| If we can go back to when you first received the letter inviting you to have your medicines reviewed, how did you feel? | Had you ever had a medication review previous to this? |
| How useful did you find the letter? | Was it informative?  Did it make sense?  Did it give you any concerns/worries?  Did it help you prepare for your medicines review? |
| Can you please describe your medicines review for me? | How did you find doing the review over the telephone?  Do you feel you had the opportunity to voice your opinion on any decisions made during the consultation?  How did you feel about the outcome of the consultation (e.g. stopping/reducing a medicine)? |
| Have you found the ‘safely stopping your medicines’ leaflet useful?  If yes, why has it been useful?  If no, what does it not include that you would have liked it to have? | What parts of the leaflet were particularly useful?  What parts weren’t? |
| During your consultation was a follow up plan agreed?  If yes, what was it about the plan that was useful?  If no, what do you think could have been done to better support you after the review? |  |
| Was a follow-up consultation held?  If yes, what was it about the consultation that was useful?  If no, what would you have liked to happen during the consultation that did not happen? | Did anything change as a result of the follow-up review? |
| Since your medicines review, how have you found stopping or reducing your medicine/s? | What sort of support did you receive from your GP/or pharmacist?  Have you continued to use your safely stopping your medicines leaflet?  If so, how have you used the leaflet and which features have been most useful?  Emotional reactions? |
| **EXPERIENCES OF STOPPING MEDICATIONS** | |
| Overall, what has the experience of stopping or reducing one of your medicines been like for you? | Positive/negative?  If positive, what contributed towards it being a positive experience?  If negative, what factors do you feel made it a negative experience?  How do you think the process could be improved? |
| Would you recommend others to think about, or speak to their GP/pharmacist about stopping or reducing one/more of their medicines? Can you explain why? |  |
| **CLOSING QUESTIONS** | |
| Is there anything else you would like to say/tell us? |  |
| Is anything else that would be helpful for you?/ Is there something else we should be doing? |  |

**Interview Guide: Staff, single interview, v1.3**

| **INTRODUCTION** | |
| --- | --- |
| - Introduce the interview, explain purpose and clarify participant’s understanding of purpose of interview. - Confirm that the participant is happy for the interview to take place, and for the interview to be audio recorded. - Reassure that it is fine if they do not want to answer any of the questions. - Talk though confidentiality, ability to withdraw. - Take consent. | |
| **Questions** | **Prompts** |
| **OPENING QUESTION** | |
| 1. First of all, can you briefly explain your role for me at the practice? | e.g. pharmacist, GP, administrative assistant |
| **ROLE IN MEDICATION MANAGEMENT** | |
| 2. What role do you have when it comes to medicines? | e.g. GP overseeing patients medicine, pharmacist reviewing medicines |
| 3. And what about your involvement when it comes to structured medication reviews? | e.g. conducting structured medicine review, administrative duties |
| 4. Prior to DEPPLOY, how did you conduct structured medicine reviews/medicine reviews? | What was the practice protocol for doing a medicines (proactive/reactive)?  What would trigger a medicines review? |
| **EVALUATING DEPPLOY** | |
| 5. What components of DEPPLOY have you engaged with as part of your role? | e.g. case-finding tool,  patient invite letter, safely stopping your medicines leaflet, feedback questionnaire |
| 6. How have you found using specific components of DEPPLOY? (Go through each that the participant has engaged with.) | What works/what does not?  What could be improved?  As an overall process, how would you evaluate its usability and effectiveness? |
| 7. In what way has DEPPLOY changed the way you conduct a review? |  |
| 8. In terms of evaluation, prior to DEPPLOY how might you have assessed whether an SMR had gone well or not? | Patient feedback?  Practice statistics? |
| 9. How would you evaluate DEPPLOY in terms of…   - workload? - cost effectiveness? - staff acceptability? - patient acceptability? |  |
| 10. What effects did DEPPLOY have on how decisions were reached with patients about reducing their medication? | Role of patient/health professional?  Shared decision making?  Role of invite letter?  Role of SSM leaflet? |
| 11. What sort of feedback, if any, have you received from patients who had their medicines reduced or stopped using DEPPLOY? | Explore why they felt patients gave this feedback.  What components did they feel patients received most benefit from?  Any specific stories/narratives of patients who have engaged with DEPPLOY? |
| 12. What is your perception of the quality of the DEPPLOY materials? | How comprehensive is the intervention? |
| 13. How important do you consider initiatives like DEPPLOY are to meet the needs of healthcare professionals and patients? | Why?  What are the incentives to use it? |
| **REFLECTING ON IMPLEMENTATION** | |
| 14. Why might there be a need for an intervention like DEPPLOY? |  |
| 15. How receptive was your organisation to implementing DEPPLOY? | Feelings of stress? Enthusiasm? Please explain. |
| 16. How do you think your organisation’s culture (general beliefs, values) impacted upon the implementation of DEPPLOY? |  |
| 17. How do you envisage DEPPLOY fitting in to your existing processes and structures related to structured medicine reviews (SMRs) and deprescribing? | Feelings about DEPPLOY being used as part of the structured medicine review programme? |
| 18. What kind of changes do you think need to be made in the practice setting for DEPPLOY to work successfully? | e.g. changes in practice,  changes in information systems, other changes |
| 19. Do you think there are any factors specific to the practice context that need to be considered when implementing DEPPLOY? | e.g. patient population,  IT systems |
| **CLOSING QUESTION** | |
| 20. Is there anything else you would like to add? | |

**Interview Guide: Senior staff, single interview, v1.3**

| **INTRODUCTION** | |
| --- | --- |
| - Introduce the interview, explain purpose and clarify participant’s understanding of purpose of interview. - Confirm that the participant is happy for the interview to take place, and for the interview to be audio recorded. - Reassure that it is fine if they do not want to answer any of the questions. - Talk though confidentiality, ability to withdraw. - Take consent. | |
| **Questions** | **Prompts** |
| **OPENING QUESTIONS** | |
| 1. Please can you outline your role for me? | *e.g. partnership level stakeholders,*  *PCN representatives* |
| 2. What role do you have around medicines? | *Explore broader level perceptions of deprescribing, e.g. its utility, current deprescribing practice.* |
| 3. What role do you have in relation to structured medication reviews? |  |
| 4. What does the term deprescribing mean to you? |  |
| **EVALUATING DEPPLOY** | |
| 5. From your perspective, what were you looking for from an intervention like DEPPLOY? | Ease of use?  Efficiency?  Patient satisfaction? |
| 6. What are the main challenges from your perspective to implementing an intervention like DEPPLOY? | Resources?  Cost? |
| 7. From your perspective, what are the main drivers for implementing an intervention like DEPPLOY? | SMR programme? |
| 8. What steps have been taken to encourage individuals to engage with DEPPLOY? | Training? |
| 9. Who are the key individuals to get on board to use DEPPLOY? | Pharmacists? GPs?  Administrative staff? |
| 10. How well did DEPPLOY fit with existing work processes and practices in your setting? |  |
| 11. In what ways, if at all, do you feel DEPPLOY will compliment/or hinder your existing structured medicine review programme? |  |
| **REFLECTING ON IMPLEMENTATION** | |
| 12. What kinds of incentives are there, if any, to help ensure that the implementation of DEPPLOY is successful? |  |
| 13. From your perspective, how compatible do you feel DEPPLOY is with current national and regional policy on Structured Medicine Reviews? |  |
| 14. How will DEPPLOY, it at all, affect your organisation’s ability to meet policy and guidance associated with Structured Medicine Reviews? |  |
| 15. How well does DEPPLOY fit with your values and norms and the values and norms of the organisation? |  |
| 16. How does DEPPLOY compare, if at all, to similar existing medicine programmes in your setting? | What advantages does DEPPLOY have over existing programmes?  What disadvantages does DEPPLOY have compared to existing programmes? |
| 17. To what extent has your organisation set goals for implementing DEPPLOY long-term? |  |
| 18. What are the goals of DEPPLOY? How and to whom will they be communicated? |  |
| **CLOSING QUESTION** | |
| 19. Is there anything else you would like to add? | |

**Interview guide: Staff, first interview (longitudinal), v1.3.**

| **INTRODUCTION** | |
| --- | --- |
| - Introduce the interview, explain purpose and clarify participant’s understanding of purpose of interview. - Confirm that the participant is happy for the interview to take place, and for the interview to be audio recorded. - Reassure that it is fine if they do not want to answer any of the questions. - Talk though confidentiality, ability to withdraw. - Take consent. | |
| **Questions** | **Prompts** |
| **OPENING QUESTION** | |
| 1. First of all, can you briefly explain your role for me at the practice? | e.g. pharmacist, GP, administrative assistant |
| **ROLE IN MEDICATION MANAGEMENT** | |
| 2. What role do you have when it comes to medicines? | e.g. GP overseeing patients medicine, pharmacist reviewing medicines |
| 3. And what about your involvement when it comes to structured medication reviews? | e.g. conducting structured medicine review, administrative duties |
| 4. Prior to DEPPLOY, how did you conduct structured medicine reviews/medicine reviews? | What was the practice protocol for doing a medicines (proactive/reactive)?  What would trigger a medicines review? |
| 5. How do you evaluate whether a review went well or not? | *Patient feedback? Practice statistics?* |
| **EXPERIENCES OF DEPRESCRIBING** | |
| 6. Moving on to focusing specifically on deprescribing. What does the term deprescribing mean to you? | *Explore connotations surrounding the term.* |
| 7. How would you describe your role in the deprescribing process? *E.g. pharmacist role vs administrative role.* |  |
| 8. How did you find the process of deprescribing? | *Did you find the process challenging/comfortable?*  *What made the process easier/difficult?*  *Emotional response to deprescribing?* |
| 9. How do you feel about the current programs/practices/and process related to structured medicine reviews and deprescribing? | *Have they been successful? If so, how have you measured this? Do you think they could be improved? What is working well/not so well?* |
| **REFLECTING ON IMPLEMENTATION** | |
| 10. Moving on to focusing on our proposed intervention (DEPPLOY). Do you think there is a strong need for an intervention like DEPPLOY? If so, why? |  |
| 11. What do you think is the general level of receptivity in your organisation to implementing an intervention like DEPPLOY? |  |
| 12. How do you think your organisations culture (general beliefs, values) that will impact the implementation of DEPPLOY? |  |
| 13. How essential do you think an intervention like DEPPLOY is to meet the needs of healthcare professionals and patients? |  |
| 14. How do you envisage DEPPLOY fitting in to the existing processes and structures related to structured medicine reviews (SMRs) and deprescribing. |  |
| 15. How do you feel about DEPPLOY being used as part of the structured medicine review programme? | Do you have any feelings of stress, enthusiasm, why? |
| 16. What kind of changes/alterations do you think you will need to make DEPPLOY work effectively in the practice setting? | e.g. Changes in practice, changes in information systems, other? |
| 17. Are there any factors specific to the practice context that need to be considered when implementing DEPPLOY? | e.g. Patient population, IT systems? |
| 18. What are your thoughts on the training you received for DEPPLOY? | What were the positive aspects of the planned training? |
| **CLOSING QUESTION** | |
| 19. Is there anything else you would like to add? | |

**Interview guide: Staff, second interview (longitudinal), v1.3**

| **INTRODUCTION** | |
| --- | --- |
| - Introduce the interview, explain purpose and clarify participant’s understanding of purpose of interview. - Confirm that the participant is happy for the interview to take place, and for the interview to be audio recorded. - Reassure that it is fine if they do not want to answer any of the questions. - Talk though confidentiality, ability to withdraw. - Take consent. | |
| **Questions** | **Prompts** |
| **EVALUATING DEPPLOY** | |
| 1. What are your thoughts on the training you received for DEPPLOY? | e.g. case-finding tool,  patient invite letter, safely stopping your medicines leaflet, feedback questionnaire |
| 2. What components of our proposed process (DEPPLOY) have you engaged with as part of your role? (e.g. case-finding tool, patient invite letter, SystmOne protocol, safely stopping your medicines leaflet, feedback questionnaire) |  |
| 3. How have you found using specified components of DEPPLOY? (Go through each that the participant have engaged with) | What works/what does not?  What could be improved?  As an overall process, how would you evaluate its usability and effectiveness? |
| 4. How well do you think DEPPLOY enables or facilitates shared decision making? |  |
| 5. How do you feel DEPPLOY integrates/complements existing processes and practices related to structured medicine reviews and deprescribing? |  |
| 6. What would you say has been the general level of receptivity towards implementing and using DEPPLOY? |  |
| 7. How well do you feel DEPPLOY has met the needs of healthcare professionals and patients? |  |
| 8. What sort of feedback, if any, have you received from patients who had their medicines reduced or stopped using DEPPLOY? | Explore why they felt patients gave this feedback.  What components did they feel patients received most benefit from?  Any specific stories/narratives of patients who have engaged with DEPPLOY? |
| 9. What is your perception of the quality of the DEPPLOY materials? | How comprehensive is the intervention? |
| 10. How do you feel about continuing to use DEPPLOY long-term? | Why?  What are the incentives to use it? |
| **CLOSING QUESTION** | |
| 11. Is there anything else you would like to add? | |
