# Supplementary material for: A consolidated framework for implementation research (CFIR) informed exploration of a primary care intervention to support deprescribing for problematic polypharmacy in older adults living with frailty (DEPPLOY) in England: a qualitative study
Source: Int J Clin Pharm. 2026 Apr 27;48(4):1594–606. doi: 10.1007/s11096-026-02140-0 (PMC13368935; doi:10.1007/s11096-026-02140-0)
Supplement: Supplementary file 4 — Supplementary file4: Additional File 4 COREQ checklist. (DOCX 32 KB) [file 11096_2026_2140_MOESM4_ESM.docx]

| **Topic** | **Guide Questions/Description** | **Detailed Information (Section, Page indicated)** |
| --- | --- | --- |
| **Domain 1: Research team and reflexivity** | | |
| *Personal characteristics* | | |
| 1. Interviewer/  facilitator | Which author/s conducted the interview or focus group? | ADO, AD and JB conducted all interviews (**Methods, Data Collection, Page 10**). |
| 2. Credentials | What were the researcher’s credentials? E.g. PhD, MD | **Author 1**: PhD **Author 2**: PhD **Author 3**: PhD **Author 4**: PhD **Author 5**: PhD **Author 6**: PhD **Author 7**: PhD **Author 8**: PhD **Author 9**: PhD **Author** 10: PhD **Author 11**: PhD A**uthor 12**: Lay leader  **Author 13:** PhD **Author 14**: PhD **Author 15:** PhD |
| 3. Occupation | What was their occupation at the time of the project? | **Author 1**: Professor of Health Service Operations **Author 2**: Senior Research Fellow **Author 3**:  Research Fellow **Author 4**: Associate Professor in Healthcare Quality and Safety **Author 5**: Professor of Healthcare Systems and Safety **Author 6**:  Associate Professor **Author 7:** Associate Professor **Author 8:** Emeritus Professor **Author 9:** Honorary Associate Professo**r** **Author 10:** Associate Professor **Author 11:** Senior Research Fellow **Author 12:** Lay leader **Author 13:** Senior Research Fellow  **Author 14:** Research Fellow **Author 15:** Professor of Medicines Use and Safety |
| 4. Gender | Was the researcher male or female? | Seven members of the research team were male and eight were female. |
| 5. Experience and training | What experience or training did the researcher have? | Author 1 is an expert in healthcare improvement with extensive experience in processes/systems analysis and qualitative publishing. She was guided in her approach to this study and data analysis by Authors 2 and 3. Authors 2 and 3 are expert qualitative methodologists who have provided extensive expertise on the use of qualitative methods in implementing and disseminating quality improvement programs in healthcare settings. Authors 4 – 7, 11 and 13-14 are experienced researchers in healthcare services and qualitative research and have collectively published numerous articles and white papers using qualitative methods.  6,8,9, 10 and 15 academic pharmacists and have extensive methods and writing experience re healthcare projects.  12 is a patient representative with extensive experience participating in healthcare research. |
| *Relationship with participants* | | |
| 6. Relationship established | Was a relationship established prior to interview commencement? | There was no prior relationship between the participants and interviewer. |
| 7. Participant knowledge of the interviewer | What did the participants know about the researcher? e.g. personal goals, reasons for doing the research | Participants received an invitation to take part in the research by post which included information on the objectives of the project. The letter was followed up with a telephone call from staff to explain the invitation letter and answer any questions (**Methods, Participants and recruitment, Page 7**). |
| 8. Interviewer characteristics | What characteristics were reported about the interviewer/facilitator?  e.g. Bias, assumptions, reasons and interests in the research topic | The research experience of the interviewers was reported (**Methods, Participants and recruitment, Page 10**). |
| Domain 2: Project design | | |
| *Theoretical framework* | | |
| 9. Methodological orientation and Theory | What methodological orientation was stated to underpin the project? e.g.  grounded theory, discourse analysis, ethnography, phenomenology, content analysis | This study took a social constructivist perspective. Data were deductively analysed using the CFIR as an a priori framework. **(*Methods, Theoretical Approach and Data Analysis, p. 8-10*)** |
| *Participant selection* | | |
| 10. Sampling | How were participants selected? e.g. purposive, convenience, consecutive, snowball | The AC-FRAIL tool was used to identify patients eligible for medication review. An invitation to an SMR consultation was sent to identified patients by post and followed up with a telephone call from staff to explain the invitation letter, answer questions and encourage uptake. A purposive sampling approach recruited patients with frailty taking anticholinergic medications recommended for adjustment or cessation.  Staff delivering the DEPPLOY intervention, and senior practice staff responsible for medicines management, were eligible for inclusion in the study. **(Methods, *Participants and Recruitment*, p. 7)** |
| 11. Method of approach | How were participants approached? e.g. face-to-face, telephone, mail, email | Post and telephone. (***Methods, Participants and Recruitment, p. 4***) |
| 12. Sample size | How many participants were in the project? | 5 patients, 6 pharmacists, 2 GPs, 2 advanced clinical practitioners, 1 practice manager 2 members of administrative staff (***Results, p. 11-12***). |
| 13. Non-participation | How many people refused to participate or dropped out? Reasons? | The recruitment method used in this study required potential participants to express interest in taking part. Therefore, no participants refused to take part because the research team did not approach potential participants in this way. However, several participants expressed an interest in taking part but could not take part in the study for the following reasons:   1. Changed mind and didn’t want to participate 2. Can’t commit to time for call 3. Hospitalisation so can’t contribute 4. Sadly passed away 5. Can’t contribute due to illness 6. Can’t contribute due to illness 7. Study recruitment had ended |
| *Setting* | | |
| 14. Setting of data collection | Where were the data collected? e.g. home, clinic, workplace | Data were recorded either on the telephone (and recorded using an encrypted recorder) or via Microsoft Teams or Zoom **(Methods, *Data Collection*, page 10).** |
| 15. Presence of non-participants | Was anyone else present besides the participants and researchers? | No, only the interviewer and participant were present for each interview. |
| 16. Description of sample | What are the important characteristics of the sample? e.g. demographic data, date | Participant gender and ethnicity were recorded.  (**Results, p.11-12**) |
| *Data collection* | | |
| 17. Interview guide | Were questions, prompts, guides provided by the authors? Was it pilot tested? | An interview guide (Additional File 3) was developed with patient and public involvement, informed by extant literature, piloted, and tailored to reflect participants’ roles and available time. The interview guide is available in **Additional File 3. (Methods, *Data Collection*, p.9-10)** |
| 18. Repeat interviews | Were repeat interviews carried out? If yes, how many? | No interviews were repeated. |
| 19. Audio/visual recording | Did the research use audio or visual recording to collect the data? | Interviews were audio recorded either via Microsoft Teams/ Zoom or using an encrypted recorder. **(Methods, *Data Collection*, p. 9-10)** |
| 20. Field notes | Were field notes made during and/or after the interview or focus group? | No field notes were made, though **Authors** would meet to debrief and share after-reflections at the conclusion of the interview. |
| 21. Duration | What was the duration of the interviews or focus group? | The interviews ranged in duration from 16 minutes to 59 minutes. **(Results, p. 11)** |
| 22. Data saturation | Was data saturation discussed? | Authors ADO, AD, LB and AL identified the repetition of participants’ insights, observations, and ideas discussed during interviews and evident in subsequent transcripts. |
| 23. Transcripts returned | Were transcripts returned to participants for comment and/or correction? | No transcripts were returned to participants for comments. |
| **Domain 3: analysis and findings** | | |
| *Data analysis* | | |
| 24. Number of data coders | How many data coders coded the data? | Four researchers (Authors ADO, AD, LB and AB) coded the data **(Methods, *Data Analysis*, p. 10)** |
| 25. Description of the coding tree | Did authors provide a description of the coding tree? | A coding framework was developed based on the constructs and domains of the Consolidated Framework for Implementation Research (CFIR). (**Methods, *Data Analysis*, p. 10).** |
| 26. Derivation of themes | Were themes identified in advance or derived from the data? | Themes were developed deductively using the CFIR framework. **(Methods, *Data Analysis*, p. 10)** |
| 27. Software | What software, if applicable, was used to manage the data? | NVivo v12^TM^ **(Methods, *Data Analysis*, p. 10)**. |
| 28. Participant checking | Did participants provide feedback on the findings? | No, participants did not provide feedback on findings. |
| *Reporting* | | |
| 29. Quotations presented | Were participant quotations presented to illustrate the themes/findings?  Was each quotation identified? e.g. participant number | Yes, participant quotations and accompanying ID are presented in both the main text **(Results, pp. 12-29).** |
| 30. Data and findings consistent | Was there consistency between the data presented and the findings? | All authors found consistency between data presented and the findings. |
| 31. Clarity of major themes | Were major themes clearly presented in the findings? | Yes, the 5 major CFIR domains are presented in both the main text (R**esults, p-12 - 29)** and Figure 2. |
| 32. Clarity of minor themes | Is there a description of diverse cases or discussion of minor themes? | Diverse cases are described throughout the findings section (**Results p.12-29**). |
